# An exploration of UK paramedics’ experiences of cardiopulmonary resuscitation-induced consciousness

**DOI:** 10.29045/14784726.2021.3.5.4.9

**Published:** 2021-03-01

**Authors:** Pete Gregory, Ben Mays, Tim Kilner, Ceri Sudron

**Affiliations:** University of Wolverhampton ORCID iD: https://orcid.org/0000-0001-9845-0920; Yorkshire Ambulance Service NHS Foundation Trust ORCID iD: https://orcid.org/0000-0002-7129-9885; University of Worcester ORCID iD: https://orcid.org/0000-0001-7725-4402; Staffordshire University ORCID iD: https://orcid.org/0000-0003-0211-0628

**Keywords:** cardiac arrest, cardiopulmonary resuscitation, consciousness, paramedic

## Abstract

**Introduction:**

Consciousness may occur during cardiopulmonary resuscitation despite the absence of a palpable pulse. This phenomenon, known as CPR-Induced Consciousness (CPR-IC), was first described over three decades ago and there has been an increase in case reports describing it. However, there remains limited evidence in relation to the incidence of CPR-IC and to practitioners’ experiences of it.

**Methods:**

A mixed-methods, cross-sectional survey of paramedics who were registered with the Health and Care Professions Council (HCPC) and working in the United Kingdom (UK) at the time of the survey. Participants who had experienced CPR-IC were asked to provide details about the number of episodes, a description of how consciousness was manifested and whether or not it interfered with resuscitation.

**Results:**

293 eligible participants completed the study and 167 (57%) said that they had witnessed CPR-IC. Of those, over 56% reported that they had experienced it on at least two occasions. CPR-IC was deemed to interfere with resuscitation in nearly 50% of first experiences but this fell to around 31% by the third experience. The most common reasons for CPR-IC to interfere with resuscitation were: patient resisting clinical interventions, increased rhythm and pulse checks, distress, confusion and reluctance to perform CPR.

**Conclusions:**

The prevalence of CPR-IC in our study was similar to that in earlier studies; however, unlike the other studies, we did not define what constituted interfering CPR-IC. Our findings suggest that interference may be related as much to the exposure of the clinician to CPR-IC as to any specific characteristic of the phenomenon itself.

## Background

Cardiopulmonary Resuscitation-Induced Consciousness (CPR-IC) is a condition whereby a patient appears to regain some level of consciousness during cardiac arrest when chest compressions are being performed, even though they have no return of spontaneous circulation (ROSC), and was first reported in the literature three decades ago (Lewinter et al., 1989). There has been an increase in the number of case reports describing the phenomenon in the intervening years (Bihari & Rajajee, 2008; Pound et al., 2017 Tobin & Mihm, 2009), but there remains limited evidence in relation to the physiological mechanism behind CPR-IC (Georgiou et al., 2014; Imberti et al., 2003), the incidence of CPR-IC (Olaussen et al., 2017) and practitioners’ experiences of CPR-IC (Olaussen et al., 2016). Of particular concern is the potential impact that CPR-IC may have on resuscitation attempts. Resuscitation guidelines advocate the provision of high quality, minimally interrupted chest compressions (Soar et al., 2015); however, work by Olaussen et al. (2016) identified that CPR-IC could compromise the delivery of effective resuscitation, thereby reducing the chances of a successful outcome. It was suggested that CPR-IC could be classified as interfering or non-interfering based upon the impact it had on the resuscitation attempt. Olaussen et al. (2016) proposed that eye opening, restlessness and agonal breathing be classified as non-interfering CPR-IC, while deliberate movements, combating rescuers and attempting to remove airway devices be classified as interfering CPR-IC. While there is little argument that physical actions, such as attempting to remove airway devices, would interfere with resuscitation, it was felt important to investigate the perceptions of individual paramedics to establish their experiences of CPR-IC and the impact these had on their resuscitation attempts. The objectives of this study were to describe patient presentations typical of CPR-IC, and to establish what features paramedics found to interfere with resuscitation.

## Methods

### Design

This was a mixed-methods, cross-sectional survey of paramedics who were registered with the Health and Care Professions Council (HCPC) and working in the United Kingdom (UK) at the time of the survey. There were no exclusion criteria.

### Questionnaire

The survey was conducted using an online survey tool (Jisc Online surveys, https://www.onlinesurveys.ac.uk/) between 8 December 2017 and 17 January 2018. The final survey was produced from several pilot questionnaires and utilised both closed questions and free-text boxes. The closed questions provided core data and allowed us to direct participants to the appropriate part of the survey, while the free-text boxes allowed participants to provide a more detailed description in their own words. Prior to commencing the survey, participants were provided with information about the survey and asked to consent to participating. We also provided an explanation of CPR-IC to help reduce the likelihood of confusion between CPR-IC and ROSC.

The initial part of the survey sought the participant’s professional background such as length of service, route to qualification and current role. All participants were asked about their current understanding of CPR-IC and where they had obtained that information (reported in Mays et al., 2019). Participants were then asked if they had experienced CPR-IC during their career. Those who answered ‘no’ exited the survey at that point, while those who answered ‘yes’ were asked to provide further details about the number of episodes, a description of how consciousness was manifested and whether or not it interfered with resuscitation. The determination of whether the CPR-IC was interfering or non-interfering was made by the participant rather than the study team. Participants were asked if the consciousness interfered with the resuscitation, and if they answered yes they were asked to provide further details of what happened and how it interfered with the resuscitation.

Where participants had experienced CPR-IC on more than one occasion there was the facility for them to describe further cases.

### Participant recruitment

The survey was promoted to paramedics via social media, the College of Paramedics (UK Paramedic Professional Body) and word of mouth. Any paramedic on the HCPC register and working within the UK at the time of the study was eligible to complete the survey, irrespective of their prior experience of CPR-IC and whether or not they currently held a patient-facing clinical role. Responding to the survey was voluntary and anonymous, with no individual identifying information being recorded.

### Data analysis

Quantitative data were analysed using descriptive statistics, while thematic analysis of free-text answers was undertaken separately by two members of the team (PG and BM) and then reviewed for concordance. For purposes of consistency, the following definition of CPR-IC was applied: signs of consciousness perceptible to the rescuer during the application of cardiopulmonary resuscitation in a patient in confirmed cardiac arrest (Mays et al., 2019). Where there was disagreement between researchers, specific cases were discussed within the research team to achieve consensus.

## Results

**Figure 1:**
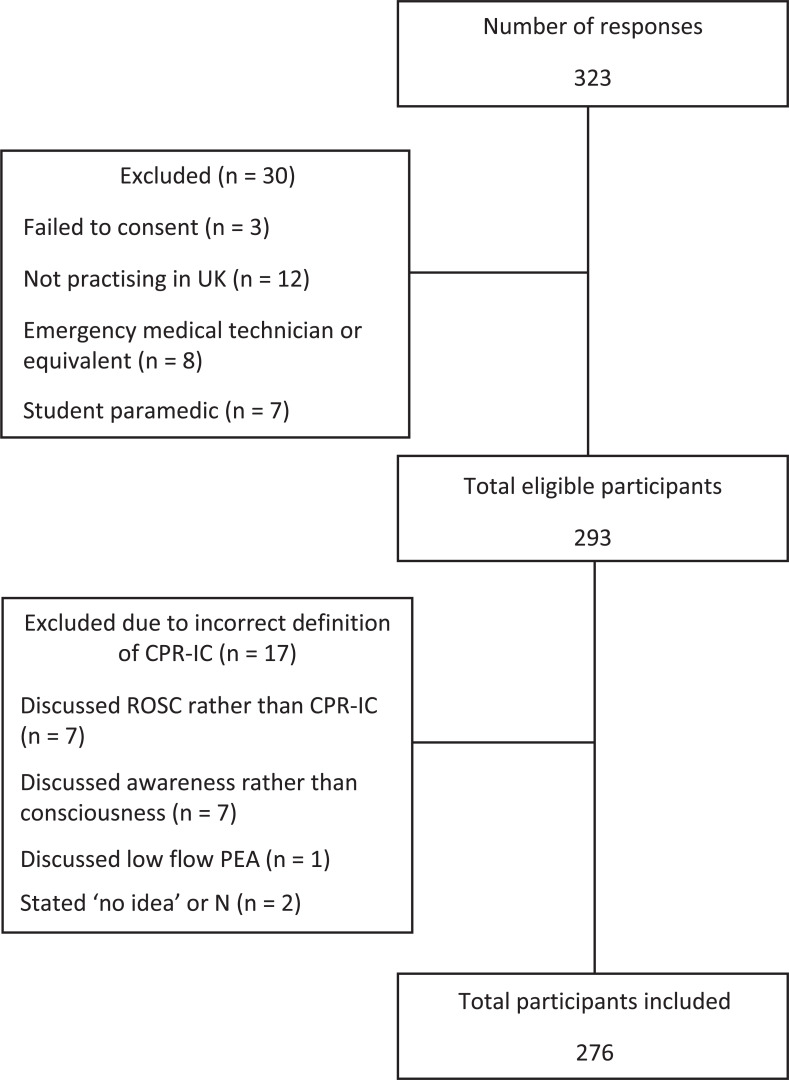
Figure 1. Study participant flowchart.

A total of 323 participants commenced the survey, of which 293 were eligible to participate (Figure 1). The participant sample represented approximately 1.17% of the total number of UK registered paramedics at the time. Table 1 shows the professional background of the study population. The majority were working in a clinical patient-facing role at the time of the survey and had more than 5 years’ experience as a paramedic. Most participants declared no specialist paramedic role or specialist qualification. In the UK, a specialist paramedic clinical role is defined by the College of Paramedics and requires a paramedic to have at least a post-graduate diploma that would normally cover primary, urgent, emergency or critical care (College of Paramedics, 2018). However, this is a fairly recent development and previously Specialist Paramedic, Emergency Care Practitioner or similar roles/titles existed without such requirements. More than half stated that they had attended in excess of 50 cardiac arrest cases during their career.

**Table 1.** Professional background of participants.

	Number (%)
**When last in regular patient-facing clinical role**	
Current	271 (92.5)
Within the last 12 months	5 (1.7)
Between 1 and 5 years ago	8 (2.7)
More than 5 years ago	9 (3.1)
**Experience as a paramedic**	
0–4 years	93 (31.7)
5–9 years	93 (31.7)
10 years or more	107 (36.5)
**Educational route to paramedic registration**	
Institute of Health and Care Development (IHCD) (or equivalent)	89 (30.4)
Certificate of Higher Education	10 (3.4)
Foundation degree/Diploma of Higher Education	132 (45.1)
BSc/BSc (Hons)	62 (21.1)
**Specialist paramedic role**	
No specialist role	191 (65.2)
Primary care	22 (7.5)
Critical care	39 (13.3)
Other	41 (14)
**Number of cardiac arrests attended**	
Up to 10	21 (7.2)
11–50	122 (41.6)
More than 50	150 (51.2)

Seventeen participants were deemed to have incorrectly defined CPR-IC (see Mays et al., 2019), so were excluded from the analysis.

One hundred and sixty-seven (57%) participants said that they had experienced CPR-IC during a resuscitation attempt, with over 56% of those reporting that they had experienced it on at least two occasions (Table 2). All participants who had experienced CPR-IC provided details of at least one case, but participants who stated that they had experienced higher numbers did not add further details for all cases.

**Table 2.** Participant’s experience of CPR-IC.

	Number (%)
Have you ever experienced CPR-IC?	
Yes	167 (57)
No	126 (43)
How many times have you experienced CPR-IC?	
Once	73 (43.7)
Twice	49 (29.3)
Three occasions	31 (18.6)
Four occasions	2 (1.2)
Five or more occasions	12 (7.2)

### Determination of consciousness

Consciousness was analysed and themed in accordance with the three domains of the Glasgow Coma Scale (GCS) (eye opening, verbal response, motor response), although many of the descriptions crossed over more than one domain. Table 3 shows the number of times that reference was made to characteristics of each domain.

**Table 3.** Signs of consciousness by patient.

	Number
**Eye opening**	
Eye opening	57
Active looking	21
	**Total 78**
Verbal	
Making noises / groaning	56
Intelligible words	6
	**Total 62**
**Motor**	
Arm movements	47
Purposeful arm movements	20
Interfering with airway devices	13
Resisting CPR	40
	**Total 120**

Cases were then themed according to whether or not the participant believed that CPR-IC had interrupted the resuscitation attempt, and comparisons were made between the descriptors. The reports of CPR-IC interfering with resuscitation diminished in those reporting second and third cases. In the first case reports, 49.7% reported that CPR-IC interfered with resuscitation, while the numbers were 47.2% for the second case reports and 30.8% for the third.

There were many similarities in the descriptions offered by respondents for both interfering and non-interfering CPR-IC; examples are provided in Table 4. The determination as to whether or not the patient’s consciousness interfered with the resuscitation was made by the participant, who indicated their perception prior to describing the event.

**Table 4.** Descriptions of consciousness: interfering and non-interfering.

**Deemed to be interfering**	**Deemed to be non-interfering**
Eyes open looking around and groaning. (Participant 349)	This patient had their eyes open and were making sounds as if typing [sic] to talk, although completely incomprehensible. (Participant 115)
Eyes open, arms moving, making noises. (Participant 395)	Arms moving. Eye following movement of people around the room. (Participant 331)
Eyes open, arms moving, making noises. (Participant 395)During chest compressions, the pt purposefully moved their arms. At one point the pt lifted their arm high enough hitting me in the face and knocking off my glasses. Eyes were open during this phase. Some form of breathing occurred – very noisy, agonal perhaps? Chest compressions were still continuing. (Participant 131)	Attempting to pull out the ETT, actively hitting out at the arms of the CPR provider and pulling her knees up and maintained a bent knee position. (Participant 756)
Eyes open, arms moving, making noises. (Participant 395)Eyes opening, speech (repeated ‘ow, ouch’ and ‘stop’ clearly a number of times) and deliberate movements – grabbing my hands (to interrupt CPR). (Participant 569)	Patient started groaning and repeatedly clearly said ‘Stop it’. I explained that I could not do that as he had just experienced a cardiac and respiratory arrest and I was keeping his heart going with the CPR and he replied ‘Bugger’ and then also said during the resuscitation ‘I’ve had enough, stop it’. (Participant 235)
Eyes open, arms moving, making noises. (Participant 395)Purposeful eye movement following people around the room. Conscious attempts to stop chest compressions. (Participant 125)	Purposeful movement, trying to push/pull at the LUCAS, movement of legs trying, patient seemed to wince at the words ‘stand clear’ prior to a shock. (Participant 996)

Thematic analysis of the descriptive data was undertaken for cases where the participant stated that the consciousness interfered with the resuscitation attempt. Responses were themed into one of six themes, which are identified in Table 5.

**Table 5.** Interference of resuscitation: themes.

What specifically interfered with the resuscitation?	Number
1. Patient resisting clinical interventions	55
2. Increased rhythm checks / paused CPR	20
3. Distress/nervousness	10
4. Confusion/distraction	15
5. Reluctance to perform CPR	3
6. Bystander concern	3

### Patient resisting clinical interventions

Patient resistance was the most commonly cited source of interference in our study and accounted for more comments than all other themes combined. Participants described a range of different experiences, with some patients interfering with resuscitation through general uncoordinated movements while others appeared more purposeful:

Pulling out airways and grabbing at staff before a shock was dangerous. (Participant 233)

Moving of head to prevent BVM being used to ventilate. Biting down on OPA when attempting to insert it. Grabbing hands of the paramedic providing chest compressions and trying to move them off his chest. (Participant 289)

### Increased rhythm checks / paused CPR

Current resuscitation guidelines recognise that clinical signs such as breathing efforts, movements and eye opening can occur during CPR and require a rhythm and pulse check as they may indicate ROSC (Soar et al., 2015). This study identified reported episodes where CPR-IC led to increased rhythm checks and pauses in chest compressions:

It was a distraction – had we achieved ROSC? I stopped compressions briefly – well the intention was briefly – so a formal check for ROSC, e.g. pulse check and rhythm check, could be made before continuing but it was very baffling to see this during a cardiac arrest. (Participant 126)

Figuring out if it was from CPR efforts or signs of ROSC. So CPR was ceased to determine if a rhythm was encountered. (Participant 294)

### Distress/nervousness

Distress and nervousness experienced by the clinician were reported, especially where the paramedics were unfamiliar with the phenomenon. One respondent said:

Patient moved head and looked directly at me! Most unnerving thing I’ve ever encountered. (Participant 58)

The event made the paramedic stop CPR, although it is unclear if they subsequently resumed when the level of consciousness deteriorated.

Another respondent discussed the disquiet felt by a fellow paramedic who was attending a case of CPR-IC. The patient was moving their arms purposefully and sitting up on occasion:

Her attempts to ‘bat’ off the hands of the compressionist, moving around, and also un-nerving the compressionist quite markedly (a fellow registered paramedic). (Participant 155)

### Confusion/distraction

Confusion and/or distraction were evident in several accounts of witnessed CPR-IC. Some respondents reflected a lack of training about CPR-IC while others were confused about how to manage a patient who was responding physically while still in cardiac arrest.

Felt put off by the patient displaying signs I had never been trained for or told about. I wasn’t sure what it was so we carried on CPR. (Participant 8)

This participant opted to continue CPR and to ignore the patient.

Distraction and traumatic to witness for responders. Required restraint of arms. (Participant 78)

In this case, a critical care doctor was on scene and so administered ketamine.

### Reluctance to perform CPR

Although not a common finding, reluctance to perform CPR on a patient who had some level of consciousness was significant because of the impact on patient care. The concern was reported on three separate occasions by different participants:

The initial Paramedic on scene was not performing CPR despite patient being in VF. It also caused some trepidation for us as a crew as to how to best proceed. (Participant 193)

### Bystander concern

Three of the participants reported resuscitation efforts being affected by the presence of bystanders when features of CPR-IC were evident:

The movement of arms up to the compressors’ hands and arms, also the situation was disturbing for all involved, including the bystanders and I feel it did interfere with the running of the arrest as we were unnerved and bystanders were questioning if he was really in arrest and his wife assumed he must be ‘getting better’, it was a conscious effort to keep focus and keep on track. (Participant 69)

## Discussion

This study set out to describe patient presentations typical of CPR-IC, and to establish what features paramedics found to interfere with resuscitation. It identified that the majority of our sample (57%) had experienced CPR-IC on at least one occasion, which is similar to the 59% reported by Olaussen et al. (2016) in their Australian-based study. Participants had experienced CPR-IC a median of two times (IQR 1-3) and the combined number of experiences exceeded 330 events of CPR-IC. The figure needs to be considered in the context of the length of clinical experience of the study participants and the high number of cardiac arrest cases that they reported they had managed during their careers. It is possible that the number of self-reported cardiac arrests may be over-estimated, especially those reporting over 50 experiences. Recent data from Yorkshire Ambulance Service (UK) showed that even their dedicated resuscitation teams only attended between 3.5 and 11.5 out-of-hospital cardiac arrests (OHCA) per year (Pilbery et al., 2019), so exposure to cardiac arrest for non-resuscitation specific paramedics is likely to be lower. This is supported by international studies which have seen mean annual OHCA exposure decline from 2.8 in 2003 to 2.1 in 2012 in Victoria, Australia (Dyson et al., 2015), while studies with higher exposure rates still report only a median of 10 per annum (Weiss et al., 2018). However, the figures for experience of CPR-IC seem reasonable as most participants reported only one (43.7%) or two (29.3%) experiences of CPR-IC and provided written narrative for their experiences.

Our study did not define the characteristics of interfering and non-interfering CPR-IC but allowed the participant to make the decision based upon their own experiences. Our findings suggest that while dichotomising CPR-IC into interfering and non-interfering may be useful, the delineation between the two categories appears fluid and may be more related to the exposure of the clinician to CPR-IC than to any specific characteristic of the phenomenon itself. The study determined that many who experienced the phenomenon for the first time considered simple eye opening to be interfering due to the disconcerting effect it had upon them, whereas in other reports purposeful motor movement and vocalisation that could be presumed to be interfering were deemed to be non-interfering by the attending paramedics. In our study, those who were more experienced in the management of CPR-IC or who had some prior understanding of the phenomenon were less likely to report as interfering those features typically associated with interfering CPR-IC (Olaussen et al., 2016). It is not clear whether this finding is directly related to increased exposure/knowledge of the phenomenon or whether it may have been a specific feature of the type of CPR-IC witnessed; however, it is important to consider the role of education in raising awareness of the phenomenon and improving care of the patient experiencing CPR-IC. An earlier paper (Mays et al., 2019) suggests that teaching about CPR-IC has not been common in UK pre-registration paramedic programmes. It appears likely that initial and ongoing education would help paramedics to better understand the phenomenon and to be less likely to be disconcerted when they first witness it. Of the six main themes identified in this study, five could be attributed to poor understanding and lack of exposure to the phenomenon. With around 29,000 Emergency Medical Service (EMS) treated cardiac arrests in England each year (Hawkes et al., 2017), there is a potential for over 260 cases of CPR-IC per annum in England alone based upon a frequency of 0.9% as reported by Olaussen et al. (2017). If the same frequency is applied to the estimated 424,000 out-of-hospital cardiac arrests in the USA each year (Go et al., 2014) and 275,000 in Europe (Atwood et al., 2005), there is a potential for nearly 6300 CPR-IC events in those regions. Given these figures, it is reasonable to suggest that initial education of all EMS providers should include CPR-IC as part of the curriculum and that CPR-IC should form part of mandatory training and continuous professional development for qualified staff.

In cases where CPR-IC was deemed to interfere with resuscitation, the most reported factor was the patient resisting clinical interventions. Where this occurs, there is clear potential for an adverse impact on the resuscitation attempt and, by extrapolation, on patient outcome. In addition to the potential impact on mortality, consideration needs to be given to physical implications such as psychological trauma and pain during the resuscitation attempt. While there is currently no international consensus or guidance for the management of a patient presenting with CPR-IC, calls have been made for sedation protocols for this group of patients. In their 2016 letter in the journal *Resuscitation*, Rice et al. (2016) argued the need for sedation in what they perceive to be a growing population, but also recognised the need for further research, education and training for pre-hospital providers. Pre-hospital sedation protocols have been implemented in some areas such as Nebraska (Rice et al., 2016) and the Netherlands (Ambulancezorg Nederland, 2014) but concern exists as to whether the use of consciousness-altering medication is appropriate. Work by Olaussen et al. (2017) found that CPR-IC was independently associated with an increased odds of survival to hospital discharge in unwitnessed/bystander witnessed events (OR 2.09, 95% CI: 1.14, 3.81; p = 0.02), but this was specifically in patients who were not given consciousness-altering medication such as midazolam, opiates and muscle relaxants. Where EMS staff witnessed the CPR-IC, it was noted that patients receiving consciousness-altering medication had poorer odds of survival compared to patients not experiencing CPR-IC (OR 0.25, 95% CI: 0.10, 0.99; p = 0.05). More work is required to understand the impact of consciousness-altering medications on patient outcome in terms of survival and well-being. It is also pertinent that many paramedic-led EMS systems do not have the legal framework or training required to undertake conscious sedation in the context of cardiac arrest.

Increased rhythm checks and pauses in chest compressions were reported by participants and may adversely impact the patient’s chances of survival. Although it is perhaps inevitable that some interruption will occur in order to check for signs of spontaneous circulation, education in the characteristics of CPR-IC may prevent the need for repeated rhythm checks and allow for early recognition of CPR-IC. The need to perform high-quality compressions and minimise interruptions is emphasised in resuscitation guidelines (Link et al., 2015; Soar et al., 2015) so interruptions caused by CPR-IC are likely to impact on patient outcome.

## Limitations

The sampling method means that there may have been bias towards those who have experienced the phenomenon, although it should be noted that 43% of our participants had never witnessed CPR-IC. The proportion of registered paramedics who responded was low, which may be reflective of the self-selecting nature of the questionnaire, but this may affect the reproducibility of the findings. It is possible that prevalence may have been over-estimated, although identifying prevalence was not the main focus of this study. Further, the study may be subject to recall bias although the critical and unusual nature of this phenomenon suggests that participants would be able to recall incidents with some clarity. It is also possible that some of the cases were not true cardiac arrest or that the patient had a ROSC rather than experiencing true CPR-IC; however, the target group of paramedics would be expected to have the ability to identify cardiac arrest, and cases where ROSC was mistaken for CPR-IC were identifiable in the narrative of the survey and removed from the analysis.

## Conclusion

In our study population, a high proportion had experienced CPR-IC, which reflects figures from other studies. If mirrored in practice it would mean that the majority of UK paramedics will attend a patient experiencing CPR-IC during their career. The perceptions of interfering and non-interfering CPR-IC were diverse and did not conform to criteria previously identified by Olaussen et al. (2016). The features that were most commonly deemed to interfere with the resuscitation were often related to the paramedic rather than the patient and could potentially be overcome with education. In order of frequency, patients resisting clinical interventions, increased rhythm checks and paused CPR, distress and nervousness of the crew, confusion and distraction, reluctance to perform CPR and bystander concern were the most reported reasons that CPR-IC was deemed to interfere with the resuscitation attempt. It is likely that initial education, international guidance and protocols on the management of CPR-IC may help practitioners to better manage patients with CPR-IC and minimise the compromise of resuscitation attempts by factors indicative of consciousness in the absence of spontaneous circulation.

## Author contributions

In accordance with ICJME guidelines, we confirm that all authors meet the following criteria for authorship:

- Substantial contributions to the conception or design of the work; or the acquisition, analysis or interpretation of data for the work; AND
- Drafting the work or revising it critically for important intellectual content; AND
- Final approval of the version to be published; AND
- Agreement to be accountable for all aspects of the work in ensuring that questions related to the accuracy or integrity of any part of the work are appropriately investigated and resolved.

PG acts as the guarantor for this article.

## Conflict of interest

None declared.

## Ethics

Ethical approval was granted by the Faculty of Education, Health and Wellbeing Ethics Panel (Health Professions, Psychology, Social Work & Social Care) at the University of Wolverhampton.

## Funding

This study was supported with a grant from the College of Paramedics Small Grants Scheme. The funder had no involvement in the study design; in the collection, analysis and interpretation of data; in the writing of the manuscript; nor in the decision to submit the manuscript for publication.
